# Lowndes County (Mississippi) Medical Society

**Published:** 1873-04

**Authors:** 


*Lowndes County (Mississippi) Medical Society.*

The officers of this Society for the present year are: J. W. M. Shattuck, M.D., President; F. H. Ervin, M.D., Vice-President; A. A. Lyon, M.D., Secretary; B. A. Vaughn, M.D., Treasurer.

Our brethren of Columbus, Mississippi, have perpetuated an able Society, and have made many valuable contributions to medical science in the past, and we doubt not will continue to do so in the future. We wish them a prosperous Society year, and trust the readers of the RECORD may hear from them often. The example of our Columbus medical friends is worthy of *practical* imitation on the part of our friends everywhere.

